# Secretory full-length human prosaposin (PSAP) inhibits SARS-CoV-2 infection through facilitating the release of S1 subunit of spike protein

**DOI:** 10.1128/mbio.00129-26

**Published:** 2026-03-26

**Authors:** Chongyang Zhang, Peiwen Ding, Xia Xiao, Bei Wang, He Huang, Qiao Zhang, Lili Ren, Zichun Xiang, Zhuo Zhou, Xiaobo Lei, Jianwei Wang

**Affiliations:** 1 NHC Key Laboratory of System Biology of Pathogens and Christophe Merieux Laboratory, National Institute of Pathogen Biology, Chinese Academy of Medical Sciences & Peking Union Medical College 220736 , Beijing, People's Republic of China; 2 State Key Laboratory of Respiratory Health and Multimorbidity, Key Laboratory of Pathogen Infection Prevention and Control (Ministry of Education), National Institute of Pathogen Biology, Chinese Academy of Medical Sciences & Peking Union Medical College 220736 , Beijing, People's Republic of China; 3 State Key Laboratory of Common Mechanism Research for Major Diseases, Suzhou Institute of Systems Medicine, Chinese Academy of Medical Sciences & Peking Union Medical College 570570, Suzhou, Jiangsu, People's Republic of China; Tsinghua University, Beijing, China

**Keywords:** SARS-CoV-2, cell entry, prosaposin (PSAP)

## Abstract

The COVID-19 pandemic, caused by severe acute respiratory syndrome coronavirus 2 (SARS-CoV-2), has profoundly impacted global public health and the economy. Systematic screening of host factors influencing viral infection is critical for understanding virus-host interactions and for developing host-targeted antiviral strategies. Here, we conducted a host cDNA overexpression screen to identify host factors involved in regulating SARS-CoV-2 replication. This approach identified prosaposin (PSAP), a precursor of lysosomal saposin activators (saposin A, B, C, and D), as an efficient host restriction factor that blocks SARS-CoV-2 entry. We demonstrate that PSAP binds with high affinity to the receptor-binding domain (RBD) of the SARS-CoV-2 spike (S) protein. Secreted PSAP efficiently inhibits infection by both SARS-CoV-2 pseudovirus and authentic virus. Mechanistically, PSAP binding facilitates the release of the S1 subunit from the S protein. Molecular docking analysis further revealed that PSAP interacts with the non-receptor-binding motif (non-RBM) region of the domain (RBD). These results suggest that PSAP inhibits SARS-CoV-2 entry through a mechanism analogous to that of neutralizing antibodies. Our findings highlight PSAP as a novel host restriction factor against SARS-CoV-2 infection, offering new insights into potential therapeutic strategies for combating SARS-CoV-2.

**IMPORTANCE:**

The systematic identification of host factors that modulate SARS-CoV-2 infection is critical for elucidating the mechanisms of virus-host interaction and for advancing the development of novel host-directed therapeutic interventions. In this study, we identify the human protein prosaposin (PSAP) as a novel and potent innate restriction factor that effectively blocks SARS-CoV-2 infection. By binding with high affinity to the spike protein’s receptor-binding domain (RBD) at a unique site, PSAP neutralizes the virus, thereby preventing cellular entry. Consequently, this discovery establishes a foundation for a novel host-directed therapeutic strategy. The development of pharmacologic agents that recapitulate PSAP’s action could yield a new class of antivirals that neutralize SARS-CoV-2 by mechanistically disrupting spike protein integrity, offering a complementary approach to conventional antibody therapies.

## INTRODUCTION

Severe acute respiratory syndrome coronavirus 2 (SARS-CoV-2), the etiological agent of COVID-19, emerged in December 2019 and triggered a devastating global pandemic (1–3). It is an enveloped, positive-sense, single-stranded RNA virus belonging to the genus Betacoronavirus. SARS-CoV-2 is the seventh known human coronavirus and the third highly pathogenic strain, following SARS-CoV and Middle East respiratory syndrome coronavirus (MERS-CoV), capable of causing severe outbreaks with significant fatality rates. In contrast, other human coronaviruses, such as HCoV-229E, HCoV-OC43, HCoV-NL63, and HCoV-HKU1, are associated with mild, seasonal respiratory illnesses (4). Since its initial emergence, SARS-CoV-2 has undergone continuous evolution, driven by widespread human transmission. This has led to the emergence of numerous variants of concern (VOC) and variants of interest (VOI). Notable VOCs, including Alpha (B.1.1.7) (5), Beta (B.1.351) (6), Gamma (P.1) (7, 8), Delta (B.1.617.2) (9, 10), and Omicron (B.1.1.529) (11), have demonstrated enhanced transmissibility, increased virulence, and/or partial immune evasion, undermining the efficacy of vaccines or prior infection-induced immunity. While several VOCs have been identified, their global impact has been comparatively limited. Critically, mutations in the viral spike protein are particularly critical, as they can enhance viral infectivity and enable escape from neutralizing antibodies.

The SARS-CoV-2 virion consists of four structural proteins: nucleocapsid (N), membrane (M), envelope (E), and spike (S) protein. The S protein is a trimeric complex embedded in the viral envelope, giving the virus its characteristic crown-like appearance. Viral entry into host cells primarily occurs through the binding of the S protein to the angiotensin-converting enzyme 2 (ACE2) receptor (12–16). The host protease furin facilitates the cleavage of S protein at a polybasic RRAP motif, producing two subunits: S1 and S2 (17). The S1 subunit consists of four domains: the N-terminal domain (NTD), the receptor-binding domain (RBD), and two C-terminal domains (CTD1 and CTD2). The RBD is responsible for binding to ACE2, initiating viral entry. After binding, the S2 subunit mediates membrane fusion between the virus and host cell. Transmembrane protease serine 2 (TMPRSS2) at the cell surface (18–20) or Cathepsin L in the endosomal cleaves the S2’ site near the fusion peptide following ACE2-mediated endocytosis (21–23). This cleavage enables the S2 subunit to form a hairpin-like structure, driving membrane fusion. The fusion peptide, rich in hydrophobic amino acids, embeds itself into the host cell membrane, facilitating the fusion process and allowing viral RNA to enter the host cell. Recent studies on the entry process of SARS-CoV-2 primarily focus on identifying both candidate receptors and inhibitory proteins or peptides. Using biotinylated SARS-CoV-2 S protein as bait, mass spectrometry analysis has identified tyrosine-protein kinase receptor UFO (AXL) as a potential receptor for SARS-CoV-2 entry into pulmonary bronchial epithelial cells (24). In addition, other receptors, such as integrin, SR-B1, Neuropilin-1, LDLRAD3 and TMEM30A, KREMEN1 and ASGR1, MYH9, KIM1, LFA1, have been suggested to promote SARS-CoV-2 entry through ACE2-dependent or independent manners (25–33). On the other hand, host restriction factors, such as LY6E, IFITM1, IFITM2, and IFITM3, have been shown to restrict SARS-CoV-2 entry (34, 35). Collectively, this diversity of entry and regulatory factors suggests that the host’s control over SARS-CoV-2 infection involves a broader array of host elements. It is plausible to postulate that the host regulates SARS-CoV-2 entry via additional, yet-to-be-identified factors beyond those already mentioned.

A functional cDNA overexpression screen is a highly sensitive method for identifying viral restriction factors and operates independently of the target gene’s baseline expression (36, 37). By enhancing the expression of specific genes, this approach can uncover potential antiviral factors or mechanisms that may not be detectable in loss-of-function studies. In this study, we identified that prosaposin (PSAP) is a restriction factor for SARS-CoV-2. We found that PSAP can be secreted into the extracellular space as a full-length protein, inhibiting the entry and infection of SARS-CoV-2. Furthermore, our findings indicate that PSAP binds to the SARS-CoV-2 spike protein through the RBD. However, PSAP does not compete with ACE2 for binding to the S1 protein *in vitro*. Instead, PSAP inhibits SARS-CoV-2 infection by facilitating the release of the S1 subunit of the spike protein. Molecular docking simulations reveal spatial interaction between PSAP and the S1 subunit, with all interaction sites located outside the RBM region of the RBD. Thus, we have identified that PSAP plays a role in inhibiting the entry and infection of SARS-CoV-2. This highlights PSAP’s potential significance in COVID-19 resistance and holds significant translational potential.

## RESULTS

### Screening of host factors involved in SARS-CoV-2 entry

To identify host factors facilitating or restricting SARS-CoV-2 entry, we conducted a functional overexpression screen using a genome-wide human complementary DNA (cDNA) library comprising 10,001 human genes (36, 37). HEK293T-ACE2 cells were transfected with individual cDNA plasmids for 24 h, followed by infection with SARS-CoV-2 pseudovirus for 48 h. Viral entry efficiency was quantified by measuring GFP reporter gene expression. *Z*-scores were calculated for each sample, with a significance threshold set at ±3 standard deviations (SD). Consistent with previous findings (34, 35), we confirmed the inhibitory effects of interferon-stimulated genes (ISGs), including LY6E, IFITM1, IFITM2, and IFITM3 on SARS-CoV-2 pseudovirus infection in 293T-ACE2 cells (Fig. S1A).

**Fig 1:**
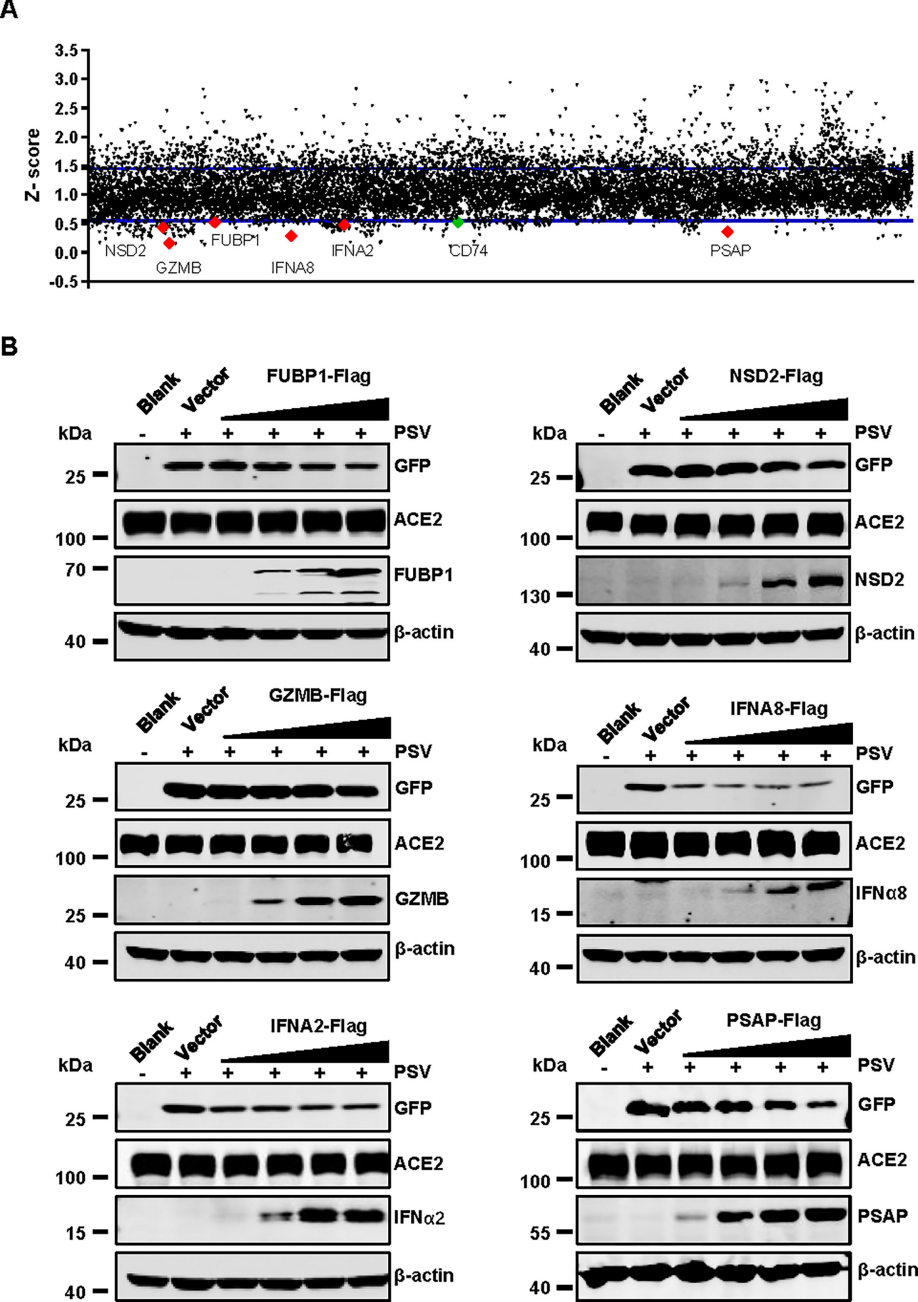
PSAP inhibits SARS-CoV-2 pseudovirus infection. (**A**) Identification of candidate genes restricting viral entry. The scatter plot shows *Z*-scores from the cDNA overexpression screen, ranked by *Z*-score. The dotted lines indicate the cutoff values. Genes with significantly different *Z*-score are highlighted. (**B**) Validation of top hits in a pseudovirus entry assay. 293T-ACE2 cells were transfected with 1 µg control vector (pCMV6-GST-Flag) or increasing doses (0.2, 0.5, 0.7, and 1 µg) of plasmids encoding FUBP1, NSD2, IFNA8, IFNA2, GZMB, and PSAP. At 36 h post-transfection, cells were then challenged with SARS-CoV-2 pseudovirus at a 4 × 10^7^ TU for 24 h. Expression levels of GFP, ACE2, Flag, and β-actin were analyzed by immunoblotting with specific antibodies.

Our screening identified several immune-related genes, including granzyme B (GZMB), interferon-alpha 2 (IFNA2), interferon-alpha 8 (IFNA8), and CD74, which significantly inhibited pseudovirus entry (Fig. 1A). Notably, CD74 has been reported to obstruct the endosomal entry pathway of coronaviruses, including SARS-CoV-2 (38). Additionally, we identified far upstream element binding protein 1 (FUBP1), PSAP, and nuclear receptor binding SET domain protein 2 (NSD2) as potent inhibitors of virus entry (Fig. 1A). Immunoblotting further validated these results, showing that FUBP1, NSD2, GZMB, IFNA8, IFNA2, and PSAP inhibited viral entry in a dose-dependent manner (Fig. 1B). Among these, PSAP, a neurotrophic precursor protein present in both secretory and integral membrane forms, stood out as a significant inhibitor of SARS-CoV-2. Given its antiviral activity, PSAP may hold significant therapeutic potential for COVID-19 treatment. This study, therefore, focuses on investigating its role in SARS-CoV-2 entry and the underlying molecular mechanisms.

### PSAP inhibits SARS-CoV-2 pseudovirus infection

**Fig 2:**
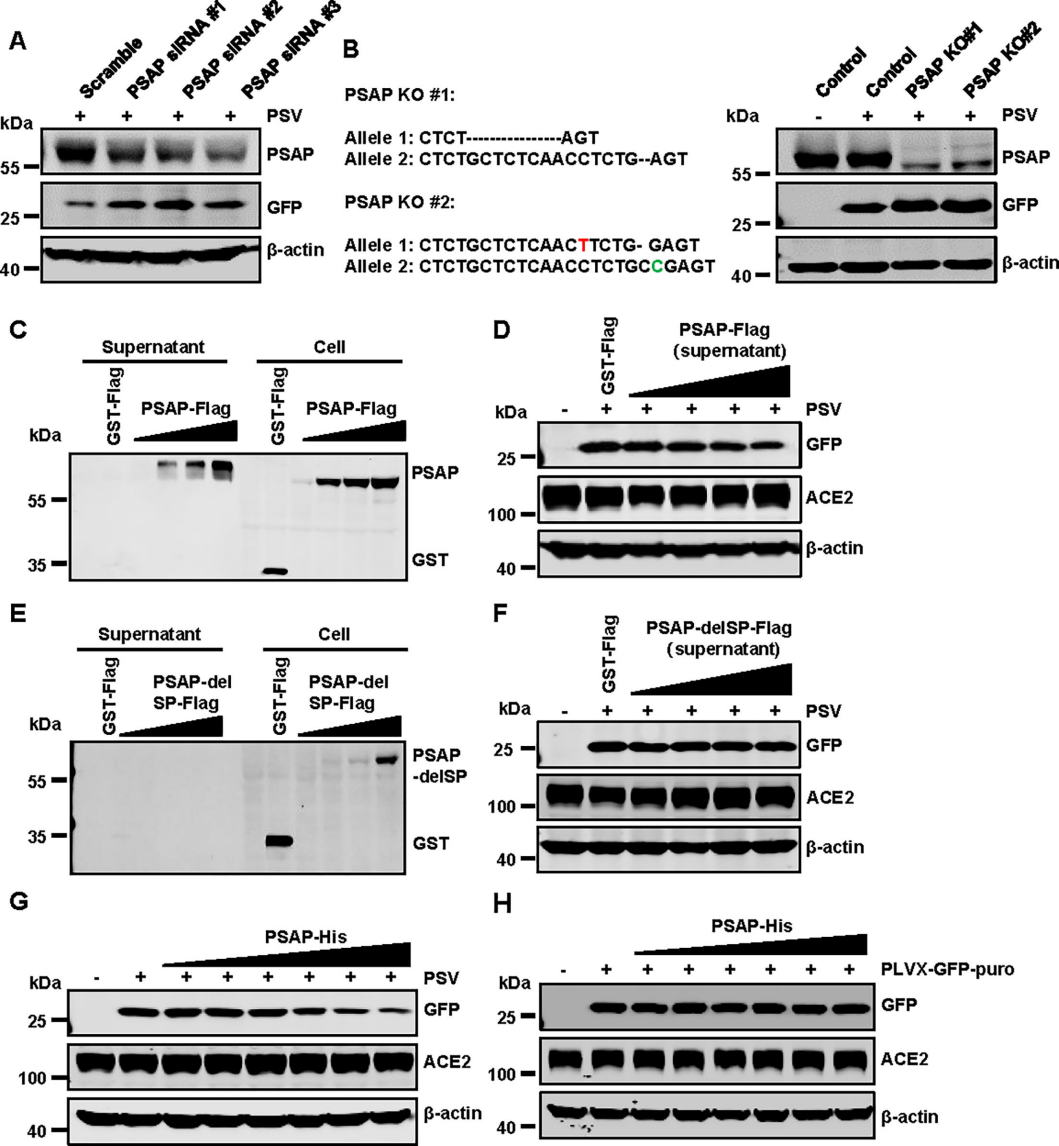
The secreted extracellular PSAP inhibits SARS-CoV-2 pseudovirus infection. (**A**) 293T-ACE2 cells were transfected with control siRNA or three independent PSAP-targeting siRNA (#1, 2, and 3) for 48 h. Cells were then challenged with SARS-CoV-2 pseudovirus. Cell lysates were analyzed by Western blotting using antibodies against PSAP, GFP, and β-actin. (**B**) Validation of PSAP knockout clones confirms its restriction activity. 293T-ACE2 wild-type and PSAP knockout cells were infected with SARS-CoV-2 pseudovirus, and lysates were analyzed by Western blotting using the indicated antibodies. (**C–F**) 293T-ACE2 cells were transfected with 1 μg of control vector (pCMV6-GST-Flag) or increasing doses (0.2, 0.5, 0.7, and 1 μg) of plasmids encoding full-length PSAP-Flag (**C**) or a signal peptide-deleted mutant (PSAP-delSP-Flag) (**E**). At 36 h post-transfection, cell lysates and culture supernatants were anzlyzed by Western blot using the indicated antibodies. Supernatants collected from the transfection in panels C and E were pre-incubated with SARS-CoV-2 pseudoviruses at 37°C for 1 h. The mixtures were then used to infect 293T-ACE2 cells respectively (**D and F**). Cell lysates were analyzed by Western blotting using the indicated antibodies. (**G and H**) Indicated concentrations of PSAP-His protein (100, 20, 4, 0.8, 0.16, 0.032, and 0 μg/mL) were incubated with SARS-CoV-2 pseudovirus or VSV-G-pseudotyped lentivirus at 37°C for 1 h. The mixtures were then used to infect 293T-ACE2 cells. Cell lysates were analyzed by Western blotting using the indicated antibodies.

As shown in Fig. 1B, PSAP protein exhibited significant inhibitory activity against SARS-CoV-2 pseudovirus entry. In contrast, no significant difference in GFP reporter gene expression was observed when cells were infected with VSV-G-pseudotyped lentivirus (Fig. S1B), indicating that the inhibition was specific to SARS-CoV-2. This inhibitory effect was not due to changes in ACE2 expression, as PSAP overexpression did not alter ACE2 levels (Fig. S1C). To determine if PSAP affected other critical host factors involved in viral entry, we analyzed the protein levels of TMPRSS2, cathepsin L (CTSL), and NRP1. The results showed that PSAP overexpression did not alter the abundance of TMPRSS2 or NRP1, nor did it reduce levels of the mature, active form of CTSL (Fig. S1D). These results demonstrate that the antiviral activity of PSAP is independent of modulating these established viral entry cofactors. To further investigate the role of endogenous PSAP, we designed three siRNAs targeting PSAP transcripts to knock down its expression. Knockdown of endogenous PSAP significantly enhanced pseudovirus entry (Fig. 2A). Additionally, we generated two PSAP knockout 293T-ACE2 cell lines using CRISPR-Cas9 gene editing (Fig. 2B). Compared to wild-type cells, PSAP knockout cells exhibited increased sensitivity to pseudovirus infection, as indicated by higher GFP reporter gene expression (Fig. 2B).

PSAP, a precursor protein for saposins, can also be secreted into various fluids (39). To confirm the presence of overexpressed PSAP in cell culture supernatants, we overexpressed PSAP in 293T-ACE2 cells and collected both supernatants and cell lysates. Western blot revealed dose-dependent PSAP expression in both fractions (Fig. 2C). To further assess the functional relevance of secreted PSAP, we incubated pseudovirus with cell supernatants at 37°C for 1 h before infecting 293T-ACE2 cells. Supernatants containing PSAP significantly inhibited pseudovirus entry (Fig. 2D). In contrast, the removal of the N-terminal signal peptide (PSAP-delSP-Flag) prevented its secretion, as it was undetectable in the supernatant (Fig. 2E), and the non-secreted PSAP protein had no effect on SARS-CoV-2 pseudovirus entry (Fig. 2F). These data confirm that secreted PSAP inhibits SARS-CoV-2 pseudovirus infection.

To further validate the inhibitory activity of the PSAP protein, we expressed and purified it using a eukaryotic system. Fivefold serial dilutions of PSAP-His protein, starting at 100 µg/mL, were co-incubated with pseudovirus at 37°C for 1 h before being added to 293T-ACE2 cells. Purified PSAP protein significantly inhibited pseudovirus infection (Fig. 2G). As controls, PSAP protein had no effect on VSV-G-pseudotyped lentivirus infection (Fig. 2H), and GST, a functionally irrelevant protein, did not inhibit pseudovirus infection (Fig. S2A).

Next, we evaluated the inhibitory activity of PSAP against different SARS-CoV-2 variants. Using a luciferase reporter system, we generated pseudoviruses of SARS-CoV-2 variants, including Alpha, Beta, Gamma, Delta, and Omicron. PSAP consistently inhibited infection by wild-type SARS-CoV-2 and all tested variants (Fig. S2B). To further assess whether PSAP inhibits the entry of other coronaviruses, we tested its effect on pseudoviruses of SARS-CoV and WIV1. PSAP significantly inhibited the entry of both viruses (Fig. S2C and D), suggesting a broader antiviral activity.

### PSAP inhibits SARS-CoV-2 infection

**Fig 3:**
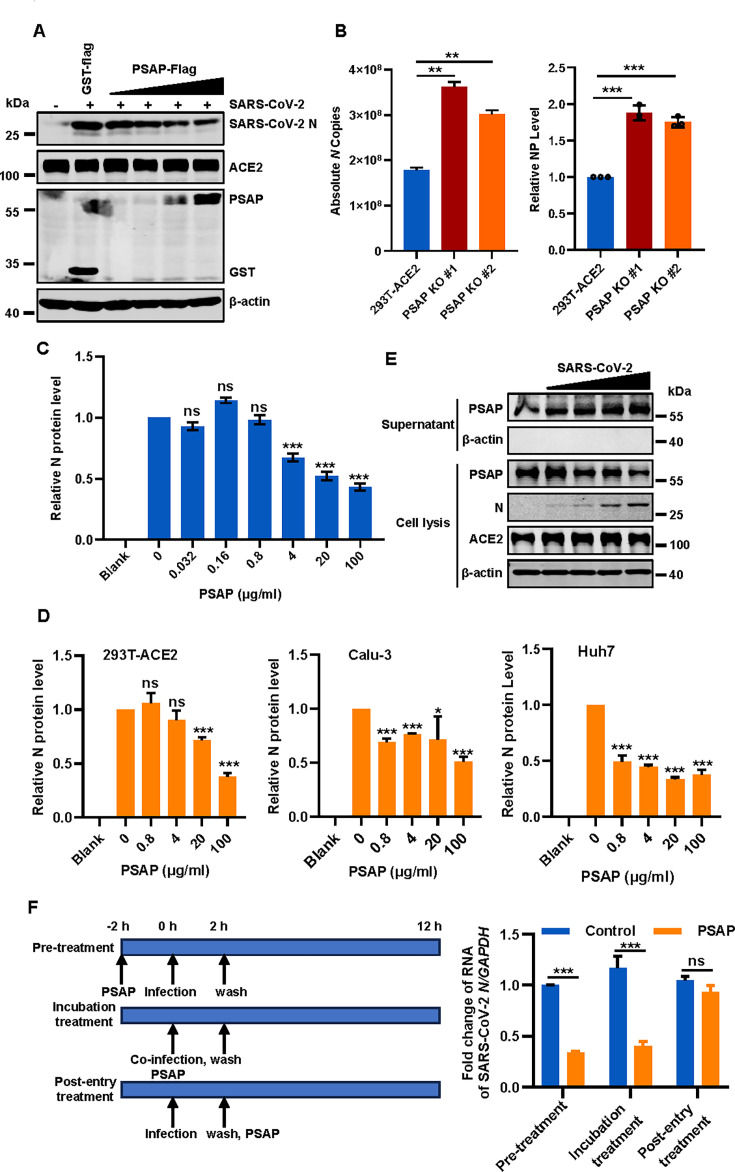
PSAP inhibits SARS-CoV-2 infection. (**A**) 293T-ACE2 cells were transfected with 1 μg of control vector (pCMV6-GST-Flag) and increasing doses of PSAP-Flag (0.2, 0.5, 0.7, and 1 μg) for 36 h and then challenged with SARS-CoV-2 authentic virus at an MOI of 0.05 for 24 h. Cells were then harvested, and Western blotting was performed using the indicated antibodies. (**B**) 293T-ACE2 wild-type or PSAP knockout cells were infected with SARS-CoV-2 at an MOI of 0.05 for 24 h. Viral *N* gene expression was measured by RT-qPCR. (**C**) Indicated concentrations of PSAP-His (0, 0.032, 0.16, 0.8, 4, 20, and 100 μg/mL) were incubated with SARS-CoV-2 at 37°C for 1 h, and then 293T-ACE2 cells were infected. Twenty-four hours later, cells were harvested, and viral *N* gene expression was quantified by RT-qPCR. (**D**) Indicated concentrations of PSAP-His protein (0,0.8, 4,20,and 100 μg/mL) were incubated with SARS-CoV-2. The mixtures were used to infect 293T-ACE2, Calu-3, and Huh7 cells. Viral *N* gene expression was quantified by RT-qPCR. (**E**) 293T-ACE2 cells were infected with different MOIs of SARS-CoV-2, and the cell supernatant and cell pellets were collected for Western blotting. (**F**) A time-of-addition assay with PSAP was performed. 293T-ACE2 cells were treated with PSAP at different stages: pre-treatment, during viral incubation, or post-entry of SARS-CoV-2 at an MOI of 0.05. The inhibitory effect was assessed by quantifying intracellular vRNA at 12 h post-infection. These experiments were repeated at least twice. ***P < 0.001; **P < 0.01; *P < 0.05; ns, *P* > 0.05.

To investigate the role of PSAP in authentic SARS-CoV-2 infection, we transfected cells with increasing amounts of a PSAP expression plasmid, followed by infection with SARS-CoV-2 at a multiplicity of infection (MOI) of 0.05. Western blot analysis revealed that PSAP overexpression reduced viral nucleocapsid (N) protein levels in a dose-dependent manner, indicating that PSAP overexpression inhibits SARS-CoV-2 infection (Fig. 3A). Conversely, to further validate the effect of endogenous PSAP on SARS-CoV-2 infection, wild-type (WT) 293T-ACE2 cells and PSAP knockout (KO) cells were infected with SARS-CoV-2 at an MOI of 0.05. Consistent with our overexpression data, PSAP-KO cells exhibited increased susceptibility to SARS-CoV-2 infection compared with wild-type controls, further supporting the antiviral role of PSAP (Fig. 3B).

Furthermore, we incubated purified PSAP-His protein with authentic SARS-CoV-2 at an MOI of 0.05 prior to infecting 293T-ACE2 cells. Consistent with our previous observations, the purified PSAP protein potently inhibited authentic SARS-CoV-2 infection in a dose-dependent manner (Fig. 3C). This inhibitory effect was confirmed across multiple relevant target cell types (Fig. 3D) and, importantly, was not attributable to cytotoxicity, as PSAP treatment did not affect cell viability (Fig. S3A). These data demonstrate that PSAP can function as a secreted, extracellular inhibitor of infection.

To investigate whether PSAP can be secreted from infected cells to exert its antiviral effect, we infected 293T-ACE2 cells with varying viral doses and analyzed PSAP distribution between cell lysates and supernatants. Intriguingly, as the MOI increased, PSAP levels in the culture supernatant rose progressively, while intracellular levels of PSAP decreased correspondingly (Fig. 3E). This suggests that SARS-CoV-2 infection actively promotes the secretion of PSAP into the extracellular environment, potentially enhancing its antiviral role. To determine whether PSAP secretion is a specific response to viral infection or a general feature of cellular stress, we evaluated its release following stimulation with the innate immune agonist poly (I:C). Unlike SARS-CoV-2 infection, poly (I:C) stimulation did not significantly induce PSAP secretion (Fig. S3B). This result confirms that PSAP secretion is a specific response to viral infection.

To pinpoint the specific stage of the SARS-CoV-2 life cycle targeted by PSAP, we performed three distinct functional assays (40): (i) pretreatment, 293T-ACE2 cells were incubated with PSAP for 2 h prior to SARS-CoV-2 attachment; (ii) co-incubation, PSAP and SARS-CoV-2 were pre-mixed and incubated *in vitro* for 2 h before being added to cells; and (iii) post-entry treatment, PSAP was added to cell 2 h after infection (Fig. 3F). Results showed that both the pretreatment and co-incubation of PSAP with the virus significantly inhibited SARS-CoV-2 replication, whereas post-entry treatment had no effect (Fig. 3F). Consistent with the pseudovirus data, these findings indicate that PSAP blocks SARS-CoV-2 infection by interfering with the viral entry stage.

### PSAP specifically interacts with the SARS-CoV-2 spike RBD

**Fig 4:**
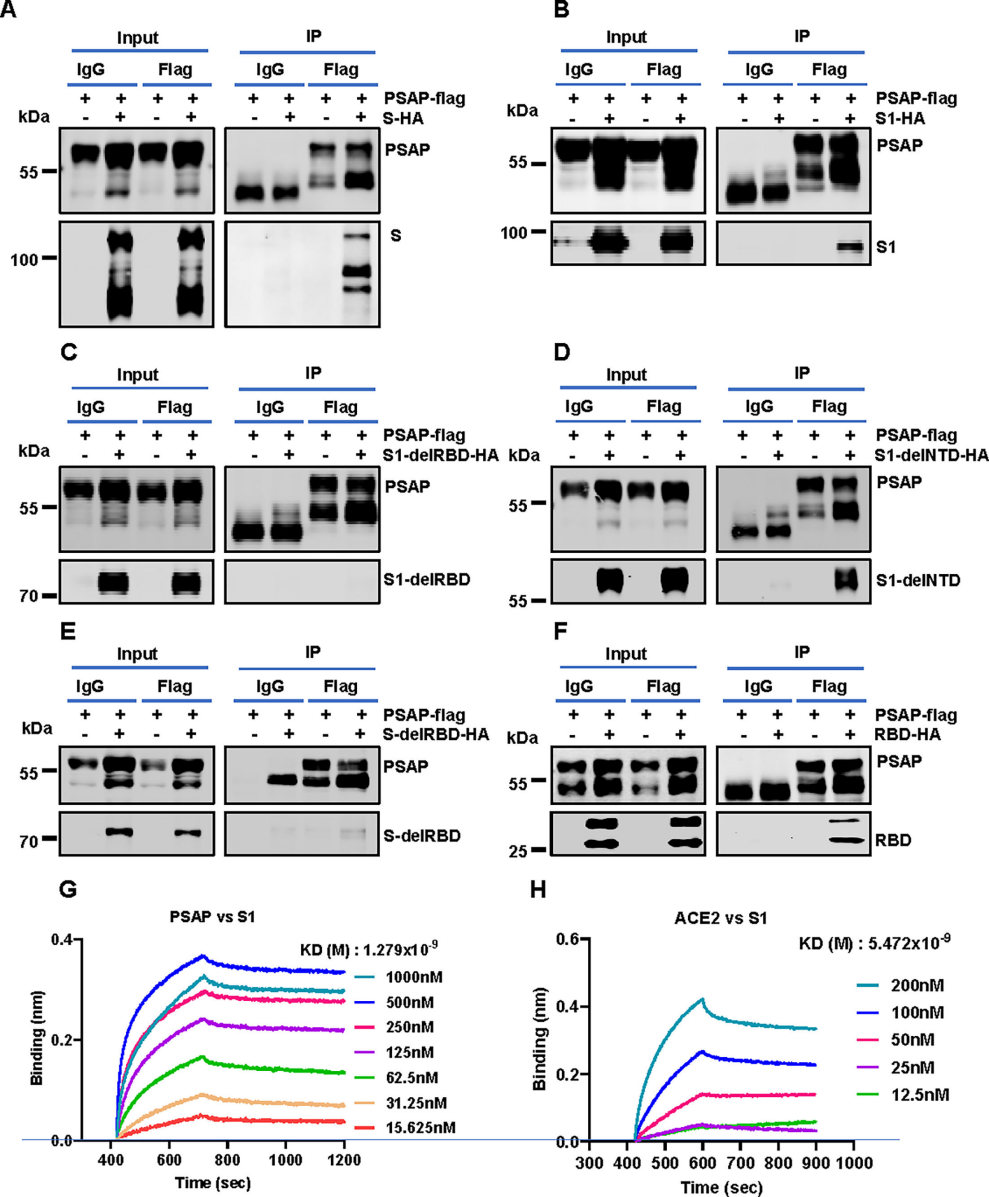
PSAP interacts with spike protein through the RBD. (**A–F**) HEK293T cells were co-transfected with plasmids expressing PSAP-Flag and spike-HA (**A**), S1-HA (**B**), S1-delRBD-HA (**C**), S1-delNTD-HA (**D**), spike-delRBD (**E**), or RBD-HA (**F**). After 36 h, cells were harvested, and lysates were subjected to immunoprecipitation with anti-Flag antibody, followed by Western blotting using the indicated antibodies. PSAP-only transfection and control mouse IgG were used as negative as control. (**G, H**) Binding of PSAP or ACE2 to SARS-CoV-2 S1 was measured by bio-layer interferometry (BLI). Serial twofold dilutions of each analyte were used for the binding assays.

To elucidate the mechanisms underlying PSAP-mediated inhibition of SARS-CoV-2 infection, we first examined whether PSAP interacts with the viral spike protein. Co-immunoprecipitation (Co-IP) assays in 293T cells co-transfected with PSAP and spike confirmed that PSAP interacts with the SARS-CoV-2 spike protein (Fig. 4A). In contrast, PSAP did not interact with ACE2 (Fig. S3C). To map the critical region within spike required for interaction with PSAP, we generated S1 and a series of S1 deletion mutants for Co-IP assays. The results showed that PSAP interacted with S1 protein (Fig. 4B), but did not bind to the RBD-deleted mutant (Fig. 4C). In addition, the deletion of the N-terminal domain (NTD) did not disrupt PSAP-spike interaction (Fig. 4D), indicating that the RBD is essential for PSAP binding. This was further confirmed by the complete loss of interaction when the RBD was deleted from full-length spike protein (Fig. 4E), as well as by direct Co-IP between PSAP and the RBD (Fig. 4F).

**Fig 5:**
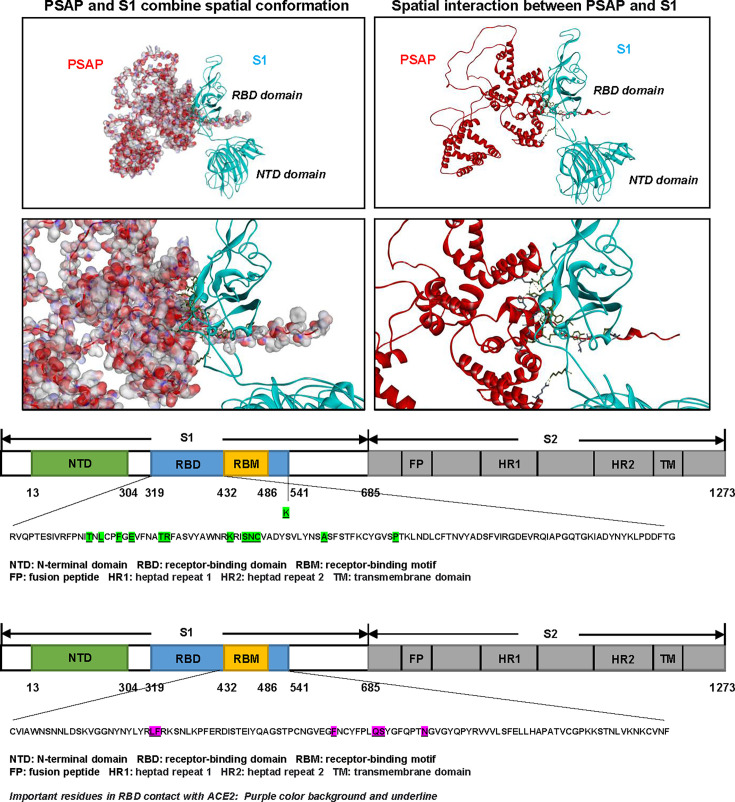
Molecular docking simulation of the PSAP-S1 interaction. Predicted binding model of PSAP (red) with the S1 subunit (blue) generated by molecular docking. Green shading on the S1 highlights residues predicted to form intermolecular contacts with PSAP. For comparison, residues on the S1 structure predicted to form contacts with ACE2 are highlighted in purple.

To quantify binding affinity, we performed biolayer interferometry (BLI) using purified proteins. Fitting to a 1:1 binding model revealed high-affinity interactions, with equilibrium dissociation constant (*K*_*D*_) of 1.279 × 10^−9^ M for SARS-CoV-2 S1 binding to PSAP (Fig. 4G) and 5.472 × 10^−9^ M for SARS-CoV-2 S1 binding to ACE2 (Fig. 4H), indicating a strong affinity between S1 and PSAP. To elucidate the structural basis of PSAP-spike interaction, we performed molecular docking simulations using Discovery Studio software based on structural data from the Protein Data Bank (PDB ID: AF_AFP07602F1 for PSAP and PDB ID: 7DDD for S1). Computational analysis revealed robust binding between PSAP and RBD, characterized by 18 distinct intermolecular contacts (Fig. 5), with the key interfacial residues listed in Table 1. These interactions included multiple hydrogen bonds, hydrophobic interactions, and salt bridges. Notably, the predicted binding interface was localized to RBD regions distinct from the receptor-binding motif (RBM), indicating that PSAP likely inhibits viral infection through a mechanism independent of direct ACE2 competition.

**TABLE 1:** Key interfacial residues and interaction modes between PSAP and S1 protein identified by molecular docking^*a*^

Amino acid interaction	Mode of action
S1: LYS529-PSAP: GLU83	Hydrogen bond
S1: LYS356-PSAP: ASP239	Electrostatic
S1: PHE338-PSAP: GLY43	Hydrogen bond
S1: THR345-PSAP: THR26	Hydrogen bond
S1: ARG346-PSAP: GLU228	Hydrogen bond
S1: ASN370-PSAP: ASP41	Hydrogen bond
S1: ALA372-PSAP: GLY17	Hydrogen bond
PSAP: GLY17-S1: TYR369	Hydrogen bond
PSAP: LYS46-S1: LEU335	Hydrogen bond
PSAP: LYS46-S1: SER359	Hydrogen bond
PSAP: LYS46-S1: ASN360	Hydrogen bond
S1: THR333-PSAP: ILE120	Hydrogen bond
S1:GLU340-PSAP: HIS47	Electrostatic
S1: CYS361-PSAP: LYS46	Hydrophobic
S1: PRO384-PSAP: LEU11	Hydrophobic
PSAP: VAL45-S1: LEU335	Hydrophobic
S1: TYR369-PSAP: ALA14	Hydrophobic
S1: TYR369-PSAP: LEU15	Hydrophobic

^*a*^ Molecular docking analysis of the PSAP– S1 binding interface identifies the key interacting amino acid residues and the specific non-covalent forces they mediate. This table is complementary to Fig. 5.

### PSAP promotes S1 subunit dissociation from spike protein to inhibit viral entry

**Fig 6:**
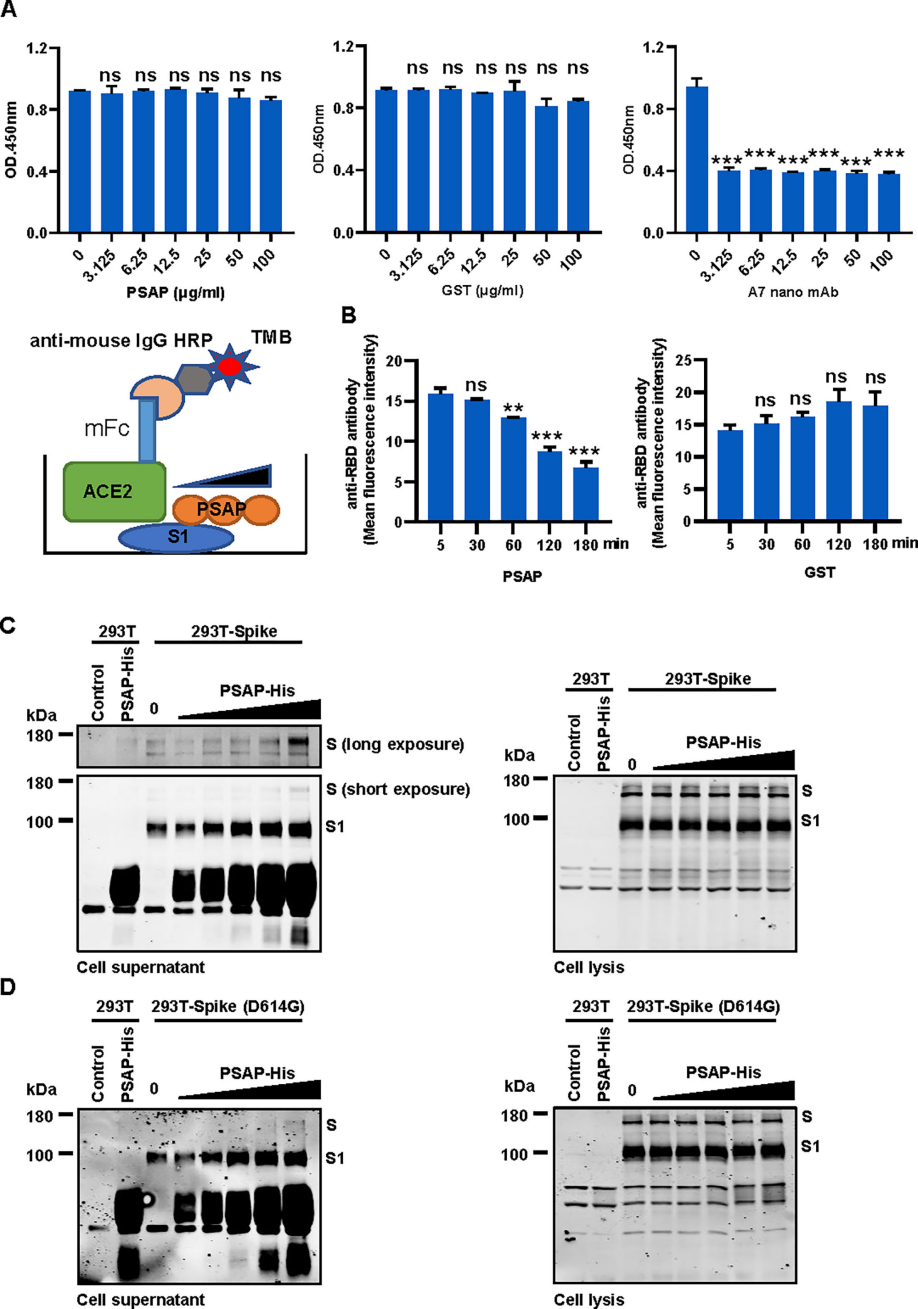
PSAP facilitates the release of the SARS-CoV-2 S1 subunit. (**A**) An *in vitro* ELISA competition experiment was performed. The coated S1-HA was incubated with PSAP-His protein, GST protein, or ACE2-mFc in different concentrations. The signal was detected by HRP-anti-mouse secondary antibody and TMB enzymatic reaction. (**B**) 293T cells stably expressing S protein were incubated with PSAP-GST or GST protein for the indicated times. Cell surface S1 subunit was detected by flow cytometry using an anti-RBD antibody. The mean fluorescence intensity of the S1 subunit on the cell membrane was shown. (**C and D**) 293T cells stably expressing the SARS-CoV-2 S protein (**C**) or S-D614G (**D**) were incubated with increasing doses of PSAP-His protein. Cell culture supernatants and cell lysates (pellet) were collected and analyzed by Western blotting to detect S1 subunit levels. These experiments were repeated at least twice. ***P < 0.001; **P < 0.01; *P < 0.05; ns, *P* > 0.05.

To further investigate the mechanism by which PSAP modulates virus entry via interaction with the spike protein, we performed an *in vitro* competition ELISA assay to assess whether PSAP competes with ACE2 for spike protein binding. A schematic diagram of the assay was shown in Fig. 6A. The results showed that PSAP had no impact on the binding between ACE2 and S1 (Fig. 6A). GST was used as a negative control, while an A7 nano antibody served as a positive control (Fig. 6A).

Next, purified PSAP protein was incubated with HEK-293T cells engineered to constitutively express the S protein. We observed a decrease in S1 subunit localization on the cell membrane, indicated by a reduction in the mean fluorescence intensity (MEI) detected by an anti-RBD antibody (Fig. 6B). Correspondingly, western blot confirmed that PSAP promotes the release of the S1 subunit into the supernatant (Fig. 6C). As a control, GST protein did not affect S1 subunit localization on the cell membrane (Fig. S4A). In contrast, S2H97, used as a positive control, also mediated the release of the S1 subunit into the supertant (Fig. S4B). The D614G mutation, known to alter RBD conformation and influence furin cleavage efficiency of the S protein (41), was evaluated by constructing 293T cells stably expressing the S-D614G protein. Similar to wild-type S protein, PSAP was also found to promote the release of the S1 subunit from the S-D614G protein (Fig. 6D). These findings indicate that PSAP inhibits SARS-CoV-2 entry by facilitating the release of the S1 subunit of the S protein, suggesting that PSAP exhibits antiviral activity comparable to that of neutralizing antibodies.

## DISCUSSION

In this study, we conducted a host cDNA overexpression screen and identified several host restriction factors, IFNα2, IFNα8, GZMB, CD74, FUBP1, NSD2, and PSAP, that inhibit SARS-CoV-2 pseudovirus entry (Fig. 1). Of the candidate host restriction factors identified in this study, IFNα2 and IFNα8 are members of the interferon-alpha (IFNα) family, which play a key role in limiting viral infection by inducing the expression of interferon-stimulated genes (42). These ISGs activate various antiviral pathways within the host cell, enhancing the cell’s resistance to viral replication and spread (43, 44). GZMB, secreted by cytotoxic CD8^+^ T cells, enhances the cytotoxic activity of these cells and contributes to the clearance of infected cells. GZMB enters target cells through perforin-formed pores, initiating apoptotic pathways that may lead to the destruction of infected cells. This function may be a key mechanism by which GZMB controls virus infection (45). CD74 p41 isoform was shown to inhibit the endosomal entry of SARS-CoV-2 by disrupting cathepsin-mediated S cleavage, which prevents viral fusion and entry (38). FUBP1 is an RNA binding protein that interacts with SARS-CoV-2 RNA (46). FUBP1 has been linked to a higher rate of SARS-CoV-2 hospitalization, with evidence suggesting that SARS-CoV-2 downregulated FUBP1 expression in the lung. This downregulation may limit FUBP1’s antiviral activity. There are currently no reports indicating that NSD2 can directly resist SARS-CoV-2 infection. However, research has demonstrated that NSD2 methylates histone marks H3K4 (H3K4Me3) and H3K36 (H3K36Me2), which enhances chromatin accessibility at the promoter of type I interferon (47). This modification facilitates the increased transcription of type I interferon, potentially contributing to its inhibitory effect on SARS-CoV-2. In addition to these genes, the role of PSAP in SARS-CoV-2 infection remains largely unexplored.

PSAP is expressed across various human tissues, including the nervous and immune systems, and plays a role in sphingolipid metabolism and lysosomal pathways (48–50). Known for its neurotrophic activity, PSAP is also associated with diseases such as Gaucher disease and metachromatic leukodystrophy. However, its potential antiviral function has not been characterized. Our results indicate that PSAP significantly inhibits SARS-CoV-2 infection and that SARS-CoV-2 infection induces the release of PSAP into the cell supernatant (Fig. 3A and E). Furthermore, the exogenous addition of purified recombinant PSAP or cell supernatant from PSAP-expressing cells effectively inhibits the entry of SARS-CoV-2 (Fig. 3C), indicating that secreted PSAP possesses antiviral properties.

While this study establishes extracellular PSAP as a direct antiviral factor, it is important to consider other biological roles of PSAP. PSAP is the precursor for four lysosomal saposins (A–D), which are essential for sphingolipid catabolism. This link to sphingolipid metabolism is particularly relevant, as sphingolipids are key components of lipid rafts, which serve as entry platforms for many viruses. Perturbations in sphingolipid metabolism—via altered saposin function—could potentially influence viral entry, replication, or assembly. However, whether exogenous PSAP treatment leads to altered intracellular processing or saposin generation is unclear, which needs further investigation.

Given its ability to inhibit SARS-CoV-2 spike-mediated pseudovirus infection (Fig. 2D), we hypothesized that PSAP interacts directly with the spike. Co-immunoprecipitation assays confirmed that PSAP binds specifically to the RBD of the SARS-CoV-2 spike protein, but with no detectable interaction between PSAP and ACE2 (Fig. 4F ; Fig. S3B). These findings suggest that PSAP may alter viral entry dynamics. Affinity measurements showed that PSAP binds to spike protein with *K*_*D*_ values in the nanomolar range (Fig. 4G), comparable to the moderate-affinity binding of 9-O-acetylated-sialic acid to the S1, suggesting a significant interaction with potential implications for viral entry inhibition (50). However, we found that this interaction does not interfere with the binding dynamics of the spike protein to ACE2.

PSAP binds to the RBD of the spike protein. However, competitive assays indicate that PSAP does not hinder the binding of the spike protein to ACE2. This raises the question of how PSAP influences virus entry. Through literature review, we note that neutralizing antibodies against SARS-CoV-2 are generally classified into four types based on their binding to specific spatial epitopes within the RBD (51). Class 1 and 2 antibodies are particularly effective at blocking the ACE2-receptor binding region located in the RBD, thereby preventing viral entry. In contrast, Class 3 antibodies typically bind to conserved domain of RBD and facilitate the release of the S1 subunit from the spike protein, thereby blocking viral fusion and entry into host cells. This observation raised the possibility that PSAP might operate through a similar mechanism. In our study, we observed that PSAP interacts with the RBD of the S protein, facilitating the release of the S1 subunit. While the precise structural mechanism is to be fully elucidated, we propose a hypothesis based on the docking model and known spike biology. Given that PSAP binds to a region of the RBD distinct from the ACE2, its interaction likely does not directly compete with receptor engagement but may instead allosterically destabilize the prefusion trimer. Binding at this alternative site could promote a conformational shift that mimics or accelerates the S1 shedding, a critical step for viral fusion.

This is supported by further analysis, which identified 18 amino acid interaction pairs between the PSAP and the S1 subunit. Notably, all interacting residues on S1 located are located outside the receptor binding motif (RBM) domain of the RBD (Fig. 5). This observation aligns with our functional data showing that PSAP inhibits virus entry without directly interfering with ACE2 binding. Therefore, we propose that the PSAP interaction with the non-RBM domain of the RBD likely induces a spatial conformational change of the spike trimer, facilitating the release of the S1 subunit. This mechanism would effectively neutralize the virus by promoting its dissociation before fusion with the host cell membrane. Future studies employing cryo-EM and site-directed mutagenesis of the predicted interface will be essential to validate this model.

The interaction mode between PSAP and the spike protein is *in vitro* critical and may require further structural analysis to elucidate the spatial details of this interaction. Such insights could deepen our understanding of PSAP’s antiviral mechanism, potentially identifying new therapeutic targets for combating SARS-CoV-2. Taken together, we demonstrate that secreted PSAP protein binds to the spike protein of SARS-CoV-2 and inhibits viral entry by promoting the release of the S1 subunit. This finding provides the first evidence that a host protein associated with the nervous system can exhibit antiviral activity. As a result, PSAP can be regarded as a restricting host factor in SARS-CoV-2 infection, presenting a novel perspective for therapeutic intervention.

## MATERIALS AND METHODS

### Cell lines

HEK293T cells were purchased from American Type Culture Collection (ATCC). HEK293T stably expressing ACE2 were generated through lentivirus transduction. Both HEK293T cells and HEK293T-ACE2 cells were cultured in Dulbecco’s modified Eagle’s medium (DMEM) (Thermo Fisher Scientific) supplemented with 10% fetal bovine serum (FBS) (Corning) at 37°C in a humidified atmosphere with 5% CO_2_. For HEK293T-ACE2 cells, the medium additionally contained 1 µg/mL puromycin.

### Viruses

The authentic SARS-CoV-2 used in this study (original strain, GenBank no. MN908947) was isolated from the throat swab of a COVID-19 patient and stored in our laboratory (3). The virus was propagated in Vero E6 cells in DMEM supplemented with 10% FBS. The TCID_50_ assay was used to determine the infectious titer of cultured SARS-CoV-2. SARS-CoV-2 pseudoviruses were obtained using a lentivirus packaging system. All experiments involving SARS-CoV-2 were conducted in the BSL-3 laboratory.

### Plasmids

A human cDNA library which contained 15,001 individual human cDNA clones was obtained from OriGene (TrueClone Collection, OriGene, Hangzhou, China). The recombinant vector encoding human PSAP transcript variant 1 (GenBank no. NM_002778.4) was cloned from cDNA library plasmid by polymerase chain reaction (PCR). Constructs including plenti-GFP, plenti-GFP/luc, pspax2, pCAGGS-spike (Original and Omicron) were stored in our laboratory, while pLV-spike plasmids from Alpha, Beta, Gamma, and Delta variants were obtained from InvivoGen. Additionally, constructs pCAGGS-S1-HA, pCAGGS-S1-delRBD-HA, pCAGGS-S1-delNTD-HA, pCAGGS-spike-delRBD were generated via homologous recombination. The lenti-PSAP-sgRNA-Hygromycin vector was cloned and modified from lentiCRISPRv2 vector (obtained from Addgene).

### Antibodies and reagents

Specific antibodies for β-actin (A1978), Flag (F3165), GFP (G6795), HA (H6908) were purchased from Sigma-Aldrich. Additional antibodies for ACE2 (A12737), β-actin (AC026) were purchased from ABclonal. The specific antibody for PSAP (10801-1-AP) was purchased from ProteinTech, while antibodies for SARS-CoV-2 N protein (40143-R019), spike protein (40591-T62) were purchased from Sino Biological. Secondary antibodies, including 680RD-labeled anti-mouse IgG (926-68072) and 800CW-labeled anti-rabbit IgG (926-32213), were purchased from LI-COR Biotechnology. Alexa Fluor 488-labeled Goat Anti-Rabbit IgG H&L antibody (ab150077) was purchased from Abcam.

The protease inhibitor cocktail (04693132001) was purchased from Roche. Protein A agarose beads (P2545) were purchased from Sigma-Aldrich, and Pierce IP lysis buffer (87788) was purchased from Thermo Fisher Scientific. S1-His (40591-V08H), ACE2-mFc (10108-H05H), RBD-hFc (40592-V02H), GST (11213-HNAE) proteins were purchased from Sino Biological.

### SARS-CoV-2 pseudovirus packaging

To package pseudoviruses, 4 µg of pspax2, 3 µg of plenti-GFP or plenti-GFP/luc, and 5 µg of pCAGGS-spike were co-transfected into 4 × 10^6^ HEK293T cells using branched polyetherimide (Sigma-Aldrich). After 8 h of transfection, the supernatant was removed, and the cells were washed with PBS before adding fresh DMEM medium. The supernatants containing pseudoviruses were collected 48 h after transfection, centrifuged, and stored at –80°C in aliquots. The transduction unit was calculated using the GFP reporter gene via flow cytometry.

### High throughput screening

Ninety-six-well plates were pre-coated with MatriGel Matrix (Corning, 354248) at 37°C for 10 min. Then, 293T-ACE2 cells were seeded at a density of 1 × 10^4^ cells per well and incubated for 12 h. Cells were transfected with plasmid (100 ng per well) using Lipofectamine 2000 (Thermo Fisher Scientific, 11668019). The supernatant was removed 24 h after transfection, and cells were infected with SARS-CoV-2 pseudoviruses for 24 h; meanwhile, 8 µg/mL polybrene was added. The supernatant was removed, and fresh DMEM medium was added into the cells for an additional 24 h. The cells were then fixed, and nuclei were stained. SARS-CoV-2 pseudovirus infection signals were assessed by calculating the immunofluorescence intensity of the GFP via high-content screening (Operetta high-content imaging system, PerkinElmer, Waltham, MA, USA) and analyzed with the associated Harmony software to calculate GFP rates. Cells transfected with an empty vector severed as the control (*y*). The GFP-positive rates in each sample (*x*) were converted to *Z*-scores (*Z*-score = *x*/average of *y*), which represent the number of standard deviations by which the GFP rates differs form the mean control value. Wells that showed significant cell toxicity (>3-fold decrease in signal) were omitted from further analysis. The cutoff value was defined as *Z*-score of ±3 SD (the number of standard deviations of the control plate value).

### Transfection of plasmids and siRNAs

Cells were transfected with plasmids or siRNA duplexes using Lipofectamine 2000 and Lipofectamine RNAiMAX (Thermo Fisher Scientific, 13778100) respectively, according to the manufacturer’s instructions. siRNAs were purchased from Guangzhou RiboBio and transfected at a final concentration of 50 nM.

### CRISPR-Cas9-mediated genome editing

PSAP gene knockout HEK293T cell lines were generated using CRISPR-Cas9 gene-editing technology. To generate PSAP knockout 293T-ACE2 cell lines, cells were transfected with a lenti-PSAP-sgRNA-hygromycin vector and selected with hygromycin (50 μg/mL) for 4 days. Cell clones were isolated, and the expression of the target gene was analyzed by western blot. The target sequence was as follows: PSAP sgRNA sequence, GCTTCTGGAGAGACTCGCAG.

### Immunoblotting

Cells were lysed on ice for 30 min in lysis buffer (25 mM Tris-Cl, 150 mM NaCl, 1 mM EDTA, 1% NP-40 or 1% Triton X-100, pH 7.4) supplemented with protease inhibitor cocktail (Roche, 04693132001). Then, the cell lysates were centrifuged at 13,600 × *g* at 4°C for 15 min to remove insoluble materials. Equal amounts of total proteins (20–100 μg) were separated by 8%–15% SDS-PAGE and then transferred to nitrocellulose membranes (Pall, 66485). After blocking with 5% nonfat dry milk solution in TBS at room temperature for 1 h, the membranes were incubated with different primary antibodies overnight at 4°C. The following day, the membranes were washed three times in TBST and then incubated with IRDye 680 or 800-labeled secondary antibodies at room temperature for 2 h. They were then scanned using the Odyssey Infrared Imaging System (LI-COR Biosciences).

### Protein expression and purification

HEK293T cells were seeded in 15 cm dishes at a density of 1 × 10^7^ per dish and incubated for 12 h. Cells were then transfected with 30 μg plasmid encoding six-histidine-tagged PSAP protein per dish, using branched polyetherimide as the transfection reagent. After 8 h, the transfection medium was removed, cells were washed with PBS, and fresh FreeStyle Expression Medium (Thermo Fisher Scientific, 12338018) was added. Supernatants containing His-tagged PSAP protein were collected 60 h post transfection, centrifuged, and purified by affinity chromatography using a Ni-NTA column (GE Healthcare), followed by further purification with a Superdex 200 Increase 10/300 size exclusion chromatograpy column (GE Healthcare). The purity of the final samples was verified by SDS-PAGE.

### Quantitative polymerase chain reaction analysis

Total RNA was extracted with TRIzol reagent and chloroform, and cDNA was synthesized using the Reverse Transcription System (Promega). Reverse transcription products were amplified with the CFX96 Real-Time System (Bio-Rad) using SYBR Green PCR Master Mix (Applied Biosystems) according to the manufacturer’s instructions, and data were normalized to the level of GAPDH in each individual sample. The 2^−ΔΔCt^ method was used to calculate relative level changes.

### Flow cytometry

ACE2 expression levels on the surface of 293T-ACE2 were detected by flow cytometry. Briefly, a specific antibody for ACE2 (1:500 dilution) was added to 4 × 10^5^ cells and incubated for 30 min at room temperature. After washing two times with PBS, cells were incubated with a 1:1,000 dilution of Alexa Fluor 488-labeled goat anti-rabbit IgG H&L antibody for 30 min at room temperature. After two washes, cells were resuspended in PBS and analyzed by FACS Canto II instrument (Becton Dickinson, Mountain View, CA).

### Co-immunoprecipitation

Cells were lysed on ice for 30 min in Pierce IP lysis buffer (25 mM Tris-HCl [pH 7.4], 150 mM NaCl, 1 mM EDTA, 1% NP-40, and 5% glycerol) supplemented with a protease inhibitor cocktail (Roche, 04693132001). The cell lysates were then centrifuged at 13,600 × *g* for 15 min at 4°C to remove insoluble materials. Equal amounts of cell lysates were incubated with the appropriate antibodies at 4°C overnight. The following day, protein A-agarose beads were washed and added to the cell lysates and incubated for an additional 3 h at 4°C. Then, immunoprecipitates were washed three times with lysis buffer. Finally, the agarose beads were boiled in 2× SDS sample buffer and analyzed by western blot with the corresponding antibodies.

### Biolayer interferometry

The binding kinetics of PSAP or ACE2 to S1 were measured with the Octet RED96e system (ForteBio, Fremont, CA, USA). All assays were conducted in standard Corning black 96-well plates, and the volume was 200 μL/well. Ligands of S1 were biotinylated using the Biotin Protein Labeling kit (Roche, 11418165001) according to the manufacturer’s instructions. Biotinylated ligands (10 μg/mL in PBS) were loaded onto streptavidin (SA) sensors (ForteBio, Germany). The PSAP or ACE2 (analyte) was diluted in a dilution series in PBS. Prior to experimentation, streptavidin sensors were hydrated in the PBS for 10 min on a shaker. The assay method for the Octet RED96e in the ForteBio Data Acquisition 12.0 program was set as follows: baseline, loading (ligands), baseline 2, association (analyte), dissociation. Data were processed on ForteBio Data Analysis 12.0 program. The *K*_*D*_ values were obtained by fitting the data.

### Statistical analysis

All data are presented as the means ± SD. Statistical analyses were performed using GraphPad Prism 8.0 software. One-way ANOVA was used to compare multiple groups, with significance indicated in the figure legends as *P < 0.05; **P < 0.01; ***P < 0.001; and ns, *P* > 0.05.

## Data Availability

All data from this study are included in the paper and are available from the corresponding author upon reasonable request.

## AFTER EPUB

*[This article was published on 26 March 2026 with errors in Acknowledgments and Funding. The errors were corrected in the current version, posted on 20 April 2026.]*

## SUPPLEMENTAL MATERIAL

Supplemental figures mbio.00129-26-s0001.pdf Figures S1 to S4.

ASM does not own the copyrights to Supplemental Material that may be linked to, or accessed through, an article. The authors have granted ASM a non-exclusive, world-wide license to publish the Supplemental Material files. Please contact the corresponding author directly for reuse.
