# Supplementary material for: Secretory full-length human prosaposin (PSAP) inhibits SARS-CoV-2 infection through facilitating the release of S1 subunit of spike protein
Source: mBio. 2026 Mar 26;17(5):e00129-26. doi: 10.1128/mbio.00129-26 (PMC13170336; doi:10.1128/mbio.00129-26)
Supplement: Supplemental figures — Figures S1 to S4. [file mbio.00129-26-s0001.pdf]

Secretory full-length human prosaposin (PSAP) inhibits SARS-CoV-2 infection through facilitating the release of S1 subunit of Spike protein

Chongyang Zhang[1#], Peiwen Ding[1#], Xia Xiao[1#], Bei Wang[1], He Huang[1,2], Qiao Zhang[1], Lili Ren[1,2], Zichun Xiang[1], Zhuo Zhou[3], Xiaobo Lei[1,2]*, Jianwei Wang[1]*

[1]NHC Key Laboratory of System Biology of Pathogens and Christophe Merieux Laboratory, National Institute of Pathogen Biology, Chinese Academy of Medical Sciences & Peking Union Medical College, Beijing, PR China;

[2]State Key Laboratory of Respiratory Health and Multimorbidity, Key Laboratory of Pathogen Infection Prevention and Control (Ministry of Education), National Institute of Pathogen Biology, Chinese Academy of Medical Sciences & Peking Union Medical College, Beijing, PR China;

[3]State Key Laboratory of Common Mechanism Research for Major Diseases, Suzhou Institute of Systems Medicine, Chinese Academy of Medical Sciences & Peking Union Medical College, Suzhou 215123, Jiangsu, PR China

[#] These authors contributed equally to this work. The author order was determined according to contribution.

*Correspondence should be addressed to X.L. （email: fyleixb@126.com） and J.W. (email: wangjw28@163.com)

Key words: SARS-CoV-2, PSAP, cell entry

Fig. S1

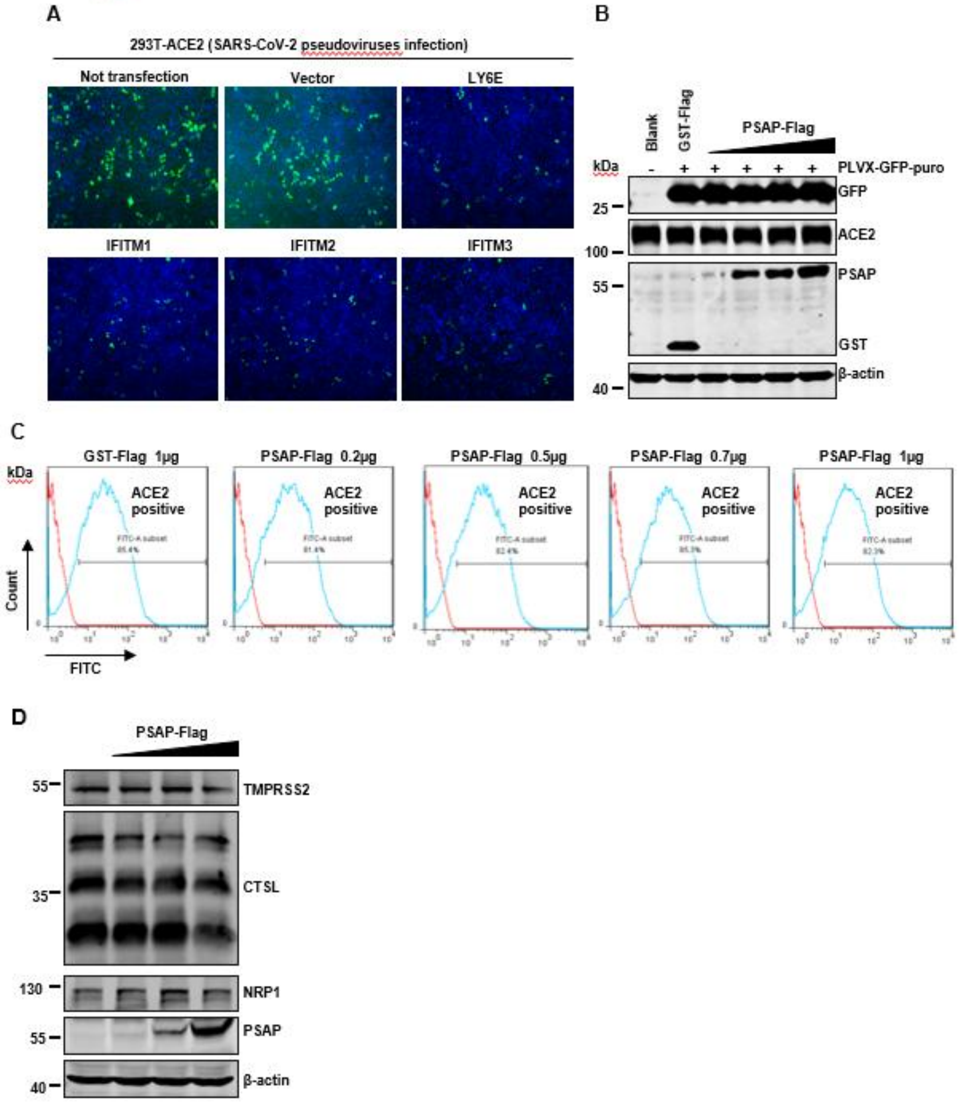

Fig. S1 PSAP did not inhibit VSVG-pseudotyped lentivirus infection. (A) 293T-ACE2 cells were transfected with 1μg of control vector (pcDNA3.1) or plasmids encoding LY6E, IFITM1, IFITM2, IFITM3 (1 μg each). At 36 h post-transfection, cells were infected with SARS-CoV-2 pseudovirus. Infection was assessed by GFP reporter expression using fluorescence microscopy). (B) 293T-ACE2 cells were transfected with the 1 μg of control vector or increasing dose (0.2, 0.5, 0.7, 1 μg) of pCMV6-PSAP-Flag. At 36 h post-transfection, cells were challenged with VSVG-pseudotyped lentivirus at a 4×107 TU for 24 h. Cell lysates were analyzed by Western blotting using antibodies against GFP, ACE2, Flag, β-actin. (C) PSAP overexpression does not alter ACE2 surface levels. 293T-ACE2 cells were transfected as described in (B). Cells were collected and analyzed by flow cytometry. ACE2 positive rates were obtained by Alexa Fluor 488 signal (Blue peak). (D) 293T-ACE2 cells were transfected with increasing amounts of plasmid encoding PSAP-Flag (0, 200, 500, 1000 ng). At 24 h post-transfection, cell lysates were analyzed by Western blotting using antibodies against Flag (PSAP), ACE2 and β-actin.

Fig. S2

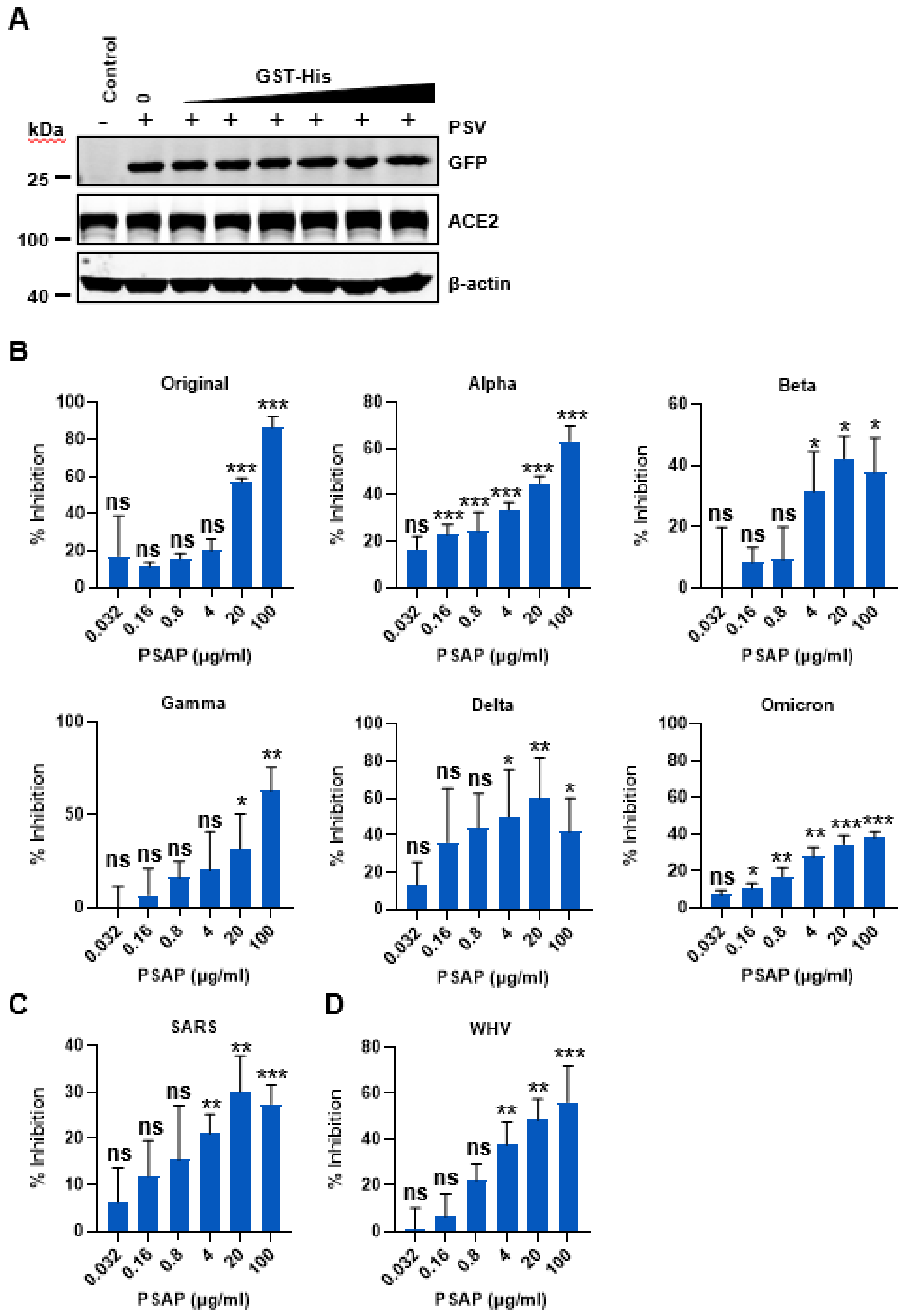

Fig. S2 PSAP inhibited distinct SARS-CoV-2 variant pseudoviruses. (A) GFP control shows no inhibition on SARS-CoV-2 pseudovirus infection. (B) PSAP inhibited SARS-CoV-2 variants infection. Indicated concentrations of PSAP-His (0, 0.032, 0.16, 0.8, 4, 20, 100 μg/ml) were preincubated with the SARS-CoV-2 variant pseudoviruses (luciferase reporter system) at 37°C for 1 h. The mixtures were then used to infect 293T-ACE2 cells. Infection was quantified by measuring luciferase activity in cell lysates. (C and D) PSAP inhibits SARS-CoV and WIV1 pseudoviruses. Different concentrations of PSAP-His protein (0, 0.032, 0.16, 0.8, 4, 20, 100μg/ml) were preincubated with

pseudoviruses bearing the spike protein of SARS-CoV (C) or WIV1 (D). The mixtures were then used to infect 293T-ACE2 cells. Cells were lysed, and luciferase activity was measured.

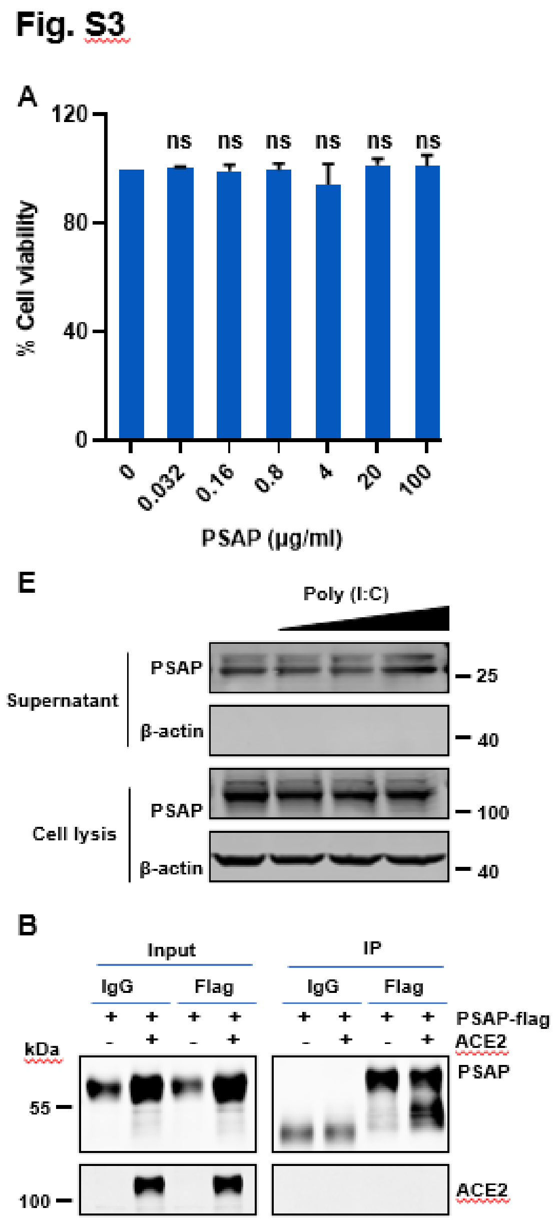

Fig. S3 PSAP protein did not affect the 293T-ACE2 cell viability and did not bind ACE2. (A) PSAP has no cytotoxic effect on 293T-ACE2 cells. 293T-ACE2 cells were incubated with the indicated concentrations of PSAP-His protein (0, 0.032, 0.16, 0.8, 4, 20, 100 μg/ml). Cell viability was assessed using the CCK8 assay. (B) 293T-ACE2 cells were transfected with increasing concentrations of poly(I:C) (0, 0.5, 1, 2μg/ml) for 6 h. Cell lysates (precipitation) and culture supernatants were analyzed by Western blotting using the indicated antibodies. (C) PSAP did not co-immunoprecipitate with ACE2. HEK293T cells were co-transfected with the pCMV6-PSAP-Flag and pXL4-ACE2 for 36 h. Cell lysates were subjected to immunoprecipitation using anti-Flag antibody or control mouse IgG.

Fig. S4

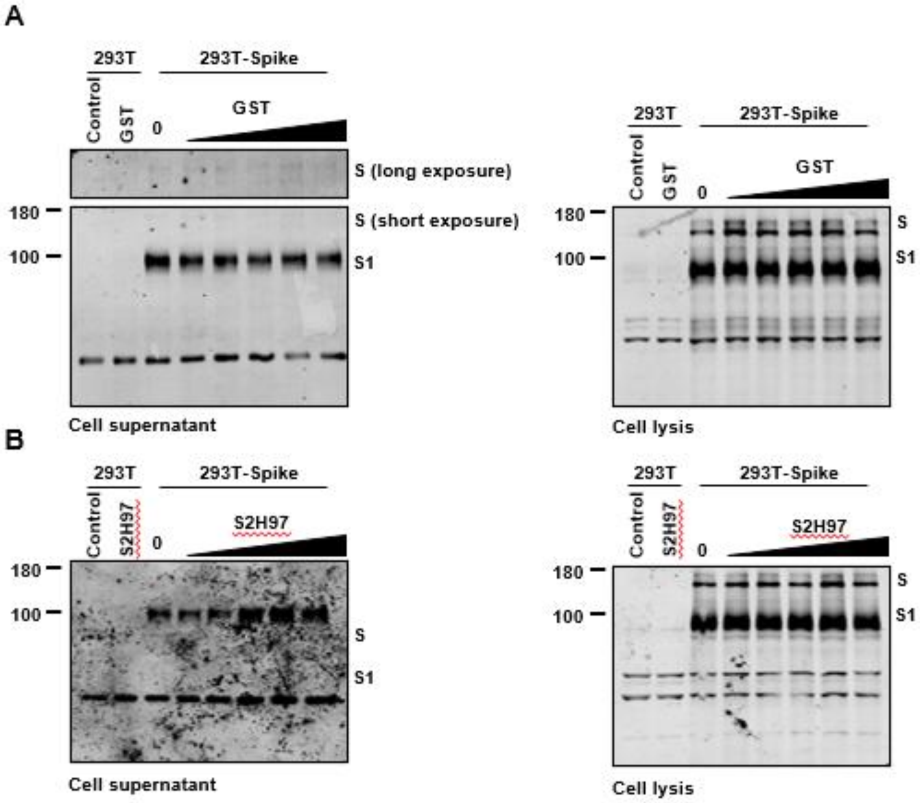

Fig. S4 S2H97 antibody promoted the release of S protein S1 subunit from the cell membrane. (A) 293T cells stably expressing the SARS-CoV-2 S protein were incubated with increasing concentrations of GST protein (0, 62.5, 125, 250, 500, 1000 μg/ml). Cell lysates and culture supernatants were analyzed by Western blotting. (B) 293T cells stably expressing the SARS-CoV-2 S protein were incubated with increasing concentrations of S2H97 antibody (0, 0.3125, 0.625, 1.25, 2.5, 5 μg/ml). Cell lysates and culture supernatants were analyzed by Western blotting.
